# Precautionary Behavior in Response to Perceived Threat of Pandemic Influenza

**DOI:** 10.3201/eid1309.070372

**Published:** 2007-09

**Authors:** M. Zia Sadique, W. John Edmunds, Richard D. Smith, William Jan Meerding, Onno de Zwart, Johannes Brug, Philippe Beutels

**Affiliations:** * Health Protection Agency, London, United Kingdom; † City University, London, United Kingdom; ‡ London School of Hygiene and Tropical Medicine, London, United Kingdom; § University Medical Centre, Rotterdam, the Netherlands; ¶ Municipal Public Health Service, Rotterdam, the Netherlands; # Antwerp University, Antwerp, Belgium

**Keywords:** Precautionary behavior, risk perception, pandemic influenza, research

## Abstract

Faced with an epidemic of an infectious disease, persons may take precautionary actions to try to reduce their risk. Such actions include avoiding situations that persons perceive to be risky, which can have negative health and economic effects. Therefore, we conducted a population-based survey of persons’ precautionary actions in response to a hypothetical influenza pandemic. For the 5 European and 3 Asian regions that had been affected by severe acute respiratory syndrome, the pattern of reported precautionary action was broadly similar across the regions; ≈75% of respondents reported that they would avoid public transportation and 20%–30% would try to stay indoors. Some regional differences were noted; Europeans were more likely than Asians to avoid places of entertainment, and Asians were more likely to avoid seeing physicians. This international survey provides insight into what might be expected during an influenza pandemic.

## Abstract 2

Public transportation was regarded as the most risky place and home as the least risky.

The risk of acquiring an infectious disease may stimulate persons to take precautionary actions to try to reduce this risk as they perceive it. The potential effect of this perceived risk–induced behavior was apparent during the outbreak of severe acute respiratory syndrome (SARS) in 2003. For example, use of public transportation in affected areas and international flights to these areas were reduced dramatically (*1*,*2*). Precautionary actions, such as avoiding public transportation or avoiding situations in which persons congregate, may have potential epidemiologic effects and would be expected to have economic consequences. The demand for certain goods and services may decline, and output may be reduced if persons avoid work or social interactions and associated purchase of goods. The economic effect of such precautionary actions may be substantial. For instance, the economic effect of SARS has been estimated at US $30–$100 billion (*3*–*5*), although the outbreak was confined to a few months and <10,000 persons were infected. Macroeconomic estimates suggest that the indirect general demand–reducing effect of SARS in nonhealth sectors was greater than the direct health effect and associated productivity losses to SARS patients and their families (*6*).

The nature and scale of this economic shock have caused concerns that pandemic influenza could have a catastrophic effect on the global economy. Understanding the factors that lead persons to take preventive actions to avoid infection may help forecast the possible course of an epidemic and its economic effect. This information would help decision-makers give appropriate advice to limit individual, community health, and economic effects.

The spread of highly pathogenic avian influenza (H5N1) and the documented illness and deaths of >300 persons in >12 countries (*7*) has heightened concerns that an influenza pandemic may be imminent. The effect of efforts to limit the dramatic health and economic consequences of such a pandemic will depend on how persons react. Research on public reaction to previous outbreaks has shown that persons may take misjudged precautionary actions (e.g., avoid places and activities that bear low risk for infection, avoid healthcare facilities for fear of infection, refuse to comply with quarantine efforts) that may contribute to the pandemic’s adverse economic effect (*8*). Therefore, to improve communication efforts by health officials, to enable pandemic containment, and to avoid unwarranted losses to the economy, knowledge of how persons will respond to the threat of an outbreak is crucial (*6*). However, research in this area is lacking (*9*). To overcome this gap, we conducted a population-based survey in 8 regions (5 European countries and 3 east Asian regions) to estimate whether persons might take precautionary actions during an influenza pandemic, the extent of such behavior, and factors that might influence it.

According to most models of health behavior, perception of being at risk is a prerequisite for behavior change, a supposition supported by empirical studies (*10*,*11*). These models endorse the belief that a high perceived risk of harm encourages persons to take action to reduce their risk. However, the direction of the association between risk perception and behavior in empirical studies varies positively, negatively, or not at all (*11*,*12*). The empirical literature that links risk perception and health behavior is subject to some debate about methods (*6*,*13*). First, the relationship between risk perception and preventive actions may be simultaneous, which makes it difficult to determine causality from observed behavior in cross-sectional data. Second, at least 2 broad methods exist for analyzing the role of risk in social science, and the choice of method is important. The most common approach is the realist approach, in which risk is seen as an objective threat or danger that can be measured independent of the social context within which it occurs (*6*,*13*). The alternative is the social constructionist approach, which describes risk as being based on objective facts about danger and hazard, amenable to rationalistic calculations, which are then mediated, perceived, and responded to in particular ways through social, cultural, and political processes (*13*). We used the social constructionist approach, in which individual and societal level factors can affect the relationship between risk perception and health behavior.

Our study sample was drawn from 2 sources: Asian regions in which SARS cases were reported and European countries in which no SARS cases occurred. Both categories could incur a new SARS or pandemic influenza outbreak. These categories are useful for determining whether previous exposure to a similar type of hazard has had any effect on risk perceptions and associated precautionary actions.

## Methods

As a part of a survey of risk perception, knowledge, and sources of information for SARS and influenza, we conducted a study on precautionary actions for a hypothetical influenza pandemic in 5 European countries (Denmark, Spain, Great Britain, the Netherlands, and Poland), and 3 Asian regions (Guangdong [People’s Republic of China], Hong Kong [Special Administrative Region, People’s Republic of China], and Singapore) that had been affected by SARS. The overall survey, from which risk behavior was analyzed separately, is described in more detail elsewhere (*14*). Brief details of the sample and questionnaire are given below.

### Sample

From September 20 through November 22, 2005, interviews were conducted in native languages by native speakers of each region, who used computer-assisted telephone interviewing and random-digit dialing. Unanswered numbers were tried again as many as 5 times; when possible, call-back appointments were made. Persons 18–75 years of age were eligible for participation, and the member of the household with the most recent birthday was invited to participate.

### Questionnaire

The questionnaire was based on a SARS and influenza risk perception questionnaire (*14*). The full questionnaire is available from www2.eur.nl/fgg/mgz/sarscontrol/questionnaire_risk_perceptions_survey.htm, and a copy of our questionnaire is available on request.

The questionnaire was translated into local languages and back translated to check the accuracy of these translations. Basic demographic and socioeconomic information about age, sex, education, health, and employment status and the like was sought. Respondents were asked to rate how serious they thought it would be to contract a range of illnesses including a heart attack, common cold, a new strain of influenza, and SARS, and how likely they themselves and the average person would be to contract these diseases in the following year. They were asked a number of questions to ascertain their level of knowledge of SARS and influenza, where they had obtained information on these diseases, and how trustworthy they perceived these information sources to be.

Respondents were then asked to imagine that a global influenza epidemic had reached their country. They were given a list of 6 places (public transportation; entertainment places such as cinemas, restaurants and theaters; shops; work or school; hospital; or home) and asked in which of these they thought they would run the greatest risk for infection. They were then randomly given 1 of 2 scenarios: a high-risk scenario in which over a 5-week period, 10% of their fellow inhabitants of all ages would be seriously ill with influenza and 0.1% would have died of the disease; and a low-risk scenario in which these rates were 2.5% and 0.025%, respectively. These scenarios were presented to the respondents in terms of rounded numbers of cases and deaths, scaled to their jurisdictions’ population size (rather than rates). Respondents were next given the following list of 8 precautionary behavior modifications and asked whether they would adopt any of them: avoid public transportation (e.g., trains, buses, airplanes); avoid going out for entertainment (e.g., bars, restaurants, theaters, cinema); limit shopping to the essentials; take leave from work; keep children out of school, even if school remains open (only adults with children were asked this question); limit physical contact with friends and family; avoid seeing doctors, even when sick from something unrelated to flu; and stay indoors at all times.

To prevent their forgetting the earlier settings on the list and to limit the interview to a maximum of 15 minutes, respondents were not given the full lists of the riskiest places and precautionary actions mentioned above. Instead, 3 places were randomly selected from the list of 6 possible places, and 3 precautionary actions were selected from the 8 possible. Additionally, the 3 places and the 3 precautionary actions were presented in random order. The main limitation of this sampling method is that it effectively reduces our sample size, but we expect sampling bias to be minimal because options (risky places and precautionary actions) were allocated randomly.

### Analysis

The analysis was performed by using STATA software version 8 (StataCorp LP, College Station, TX, USA). Simple *t* test was used to compare the differences in means (or proportions) between the 2 broad sources of samples (Europe and Asia) in terms of riskiest place and adopting precautionary behavior. Probit regression was used to assess the effect of individual- and regional-level covariates on each reported precautionary action. The main outcome variable was whether respondents reported that they would avoid the places presented to them. For 8 different specifications, the explanatory variables remain the same and only the outcome of interest (the probability of taking the preventive action) varied. We did not adjust for multiple comparisons, which should be considered when interpreting these results.

For the regression analysis, we controlled for respondents’ age, sex, region of residence, educational history, and perceived risk for influenza. Our measure of perceived risk was based on protection motivation theory (*15*), which proposes that the intention to protect oneself depends on 4 factors: 1) perceived severity of a threatened event, 2) perceived probability of the occurrence (vulnerability), 3) perceived efficacy of the recommended preventive behavior (perceived response efficacy), and 4) perceived self-efficacy (level of confidence in one’s ability to undertake the recommended preventive behavior). Risk perception (beliefs about potential harm) has many dimensions, but in keeping with nearly all theories, we focused on only 2 (*12*): 1) likelihood and 2) severity of harm if no action is taken.

We also examined the added effect of response efficacy and self-efficacy on precautionary actions. To measure persons’ perceived probability of harm/infection (vulnerability) we asked respondents, “How likely do you think it is that you will develop or contract flu from a new flu virus in the case of global flu outbreak?” For severity, we asked, “How serious would it be for you to get the disease in the next year?” In line with protection motivation theory (*14*,*15*), risk perception was constructed by multiplication of severity (scale of 1 to 10) and vulnerability (scale of 1 to 5) scores. To make the severity and likelihood scores comparable, the severity score was first divided by 2. To normalize the skewed distribution of the constructed risk perception variable, a square-root transformation was performed, which resulted in a measure of risk perception on a scale from 1 (low) to 5 (high) (*14*).

## Results

### Respondents

Of the eligible persons who were contacted by phone, 42% completed the interview and the rest refused to participate, which resulted in a sample size of 3,436. The cooperation rate varied between 21% in the United Kingdom and 81% in Poland (*14*). Unadjusted summary statistics and description of the key variables of interest are given in Table 1. The number of respondents in each participating region ranged from 401 to 502 (Table 2). Table 1 shows that respondents from European countries had a higher perceived risk for influenza, lower perceived risk for SARS, and in general were older than respondents from Asia. Compared with Asians, relatively more Europeans had a secondary education, fewer had a university education, and substantially fewer lived in urban areas.

**Table 1:** Variables measured in survey of risk perception for pandemic influenza, Europe and Asia*

Variable	Definition	Mean score (SD)	
		Respondents from Europe (n = 2,196)	Respondents from Asia (n = 1,240)
SARS risk perception	Risk perception score (1–5)	2.47 (0.95)	2.95 (1.13)
Influenza risk perception	Risk perception score (1–5)	2.95 (1.01)	2.83 (1.05)
Influenza severity	Perceived severity of influenza (1–10)	6.94 (2.55)	6.56 (2.69)
Influenza vulnerability	Perceived vulnerability to influenza infection (1–5)	2.81 (1.17)	2.75 (1.24)
Sex	1 if female	0.40 (0.49)	0.45 (0.50)
Age	Age in years	47.46 (14.32)	39.13 (15.03)
Education medium	1 if respondent has more than a secondary but at least a higher secondary education	0.59 (0.49)	0.51 (0.50)
Education high	1 if respondent has university qualification	0.30 (0.46)	0.42 (0.49)
Urban area	1 if respondent’s area of residence is city/town	0.61 (0.49)	0.96 (0.20)
European region	1 if respondent is from European region	NA	NA
High-risk scenario	1 if given outbreak scenario is high risk	0.50 (0.50)	0.66 (0.47)
Health	Health status on 1–6 Likert scale	4.28 (1.10)	4.25 (1.08)
Vaccinated	1 if vaccinated against influenza in past year	0.19 (0.39)	0.22 (0.41)
Employed	1 if employed	0.60 (0.49)	0.60 (0.49)

*NA, not available.

**Table 2:** Perceived risk of setting during influenza pandemic, Europe and Asia*

Location	Sample size	Most risky†	Least risky†
Guangdong, PRC	409	Entertainment (56)	Home (0)
Hong Kong, SAR, PRC	401	Public transportation (52)	Home (2)
Singapore	430	Entertainment (48)	Home (12)
Spain	427	Public transportation (63)	Home (3)
Poland	502	Public transportation (60)	Home (1)
Denmark	463	Public transportation (58)	Home (4)
Great Britain	401	Public transportation (49)	Home (4)
The Netherlands	403	Public transportation (48)	Home (5)

*PRC, People’s Republic of China; SAR, Special Administrative Region. †Numbers in parentheses indicate percentage of respondents who were given this option.

### Riskiest Place

**Figure 1:**
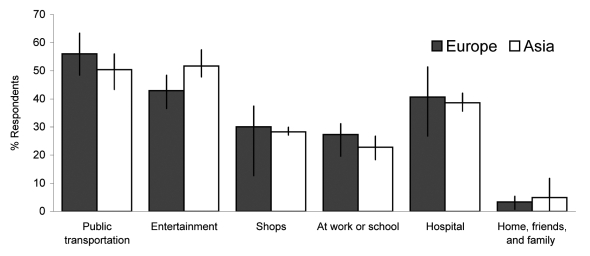
Proportion of respondents who reported each setting as the riskiest for acquiring pandemic influenza, by region. Vertical lines indicate range of means for each region.

Public transportation was identified as the riskiest place by >54% of persons who were given this option (43% in Singapore to 63% in Spain; Table 2) and by respondents from 6 of the 8 regions. Places of entertainment were generally ranked as the next most risky setting (in China and Singapore the ranking of public transportation and entertainment was reversed), followed by hospitals, shops, then work or school (Figure 1). Respondents from all regions reported the home to be the least risky setting (Table 2, Figure 1).

### Precautionary Behavior

**Figure 2:**
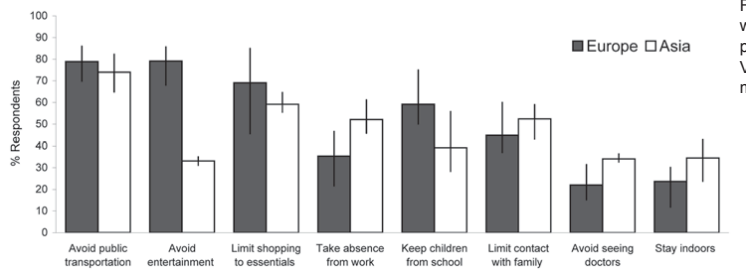
Proportion of respondents who reported that they would take precautionary actions, by region. Vertical lines indicate range of means for each region.

Avoidance of public transportation was consistently reported across the region as the most likely precautionary behavior. From 65% (in Singapore) to 85% (in Great Britain) of respondents reported that they would avoid public transportation. Similar proportions of European respondents reported that they would avoid places of entertainment, although a far smaller proportion of Asian respondents said that they would (Figure 2), despite Asians being more likely to report this setting as risky (Figure 1). Approximately 60% of respondents said that they would shop for essentials only, and ≈50% said that they would take leave from work, prevent their children from attending school, or limit contact with friends and family. Approximately 25% of European and 35% of Asian respondents said that they would try to stay indoors or avoid seeing physicians (Figure 2). Univariate analysis results suggested a statistically significant difference between regions in terms of proportions of persons who would adopt precautionary actions in case of a hypothetical influenza outbreak (Table 3).

**Table 3:** Perception of risk for hypothetical pandemic influenza, Europe and Asia

Variable	Sample size*	Europe, % (SD)	Asia, % (SD)	H_0_: proportions (*E*) – proportions (*A*) = 0†
Riskiest place				
Public transportation	1,700	56 (0.50)	50 (0.50)	0.0234
Entertainment	1,793	43 (0.50)	52 (0.50)	0.0004
Shop	1,702	30 (0.46)	28 (0.45)	0.4340
Work/school	1,668	27 (0.45)	23 (0.42)	0.0444
Hospital	1,749	41 (0.49)	39 (0.49)	0.4025
Family	1,696	3.4 (0.18)	5 (0.22)	0.1190
Precautionary behavior				
Avoid public transportation	1,341	79 (0.41)	74 (0.44)	0.0418
Avoid entertainment	1,263	79 (0.41)	33 (0.47)	0.0000
Limit shopping to essentials	1,355	69 (0.46)	59 (0.49)	0.0002
Be absent from work	1,307	35 (0.48)	52 (0.50)	0.0000
Keep children from school	349‡	59 (0.49)	39 (0.49)	0.0002
Limit contact with friends/family	1,293	45 (0.50)	52 (0.50)	0.0100
Avoid seeing doctors	1,310	22 (0.42)	34 (0.47)	0.0000
Stay indoors	1,316	24 (0.43)	35 (0.48)	0.0000

*Sample size represents the number of persons randomly chosen to answer each option. †H_0_, null hypothesis that difference between European (*E*) and Asian (*A*) proportions is not statistically significant. Significant differences using p value of *t* test. ‡This question was asked of only the 349 respondents who had a child going to school.

Multivariate regression was used to test the association between the likelihood of reporting precautionary actions and individual-, country-, and regional-level characteristics (Table 4). The coefficients in Table 4 reflect marginal effects, which can be interpreted as probabilities. For example, the coefficient attached to the European region in the first regression equation (avoiding public transportation) was 0.038, which can be interpreted as Europeans being 3.8% more likely than Asians to avoid public transportation.

**Table 4:** Results of regression analysis (marginal effects) of precautionary behavior for hypothetical influenza pandemic, Europe and Asia

Variable	Avoid public transportation	Avoid entertainment	Limit shopping	Take absence from work	Keep children from school	Limit contact with family/ friends	Avoid seeing doctor	Stay indoors
Sex (male)	0.005	–0.048	–0.054*	0.042	0.017	–0.021	–0.018	0.013
Age	–0.001	0.003*	0.001	–0.003*	–0.004	0.001	–0.001	0.001
Education medium	0.039	0.113*	0.175	–0.023	–0.223*	–0.015	–0.015	–0.033
Education high	0.033	0.139*	0.185*	–0.030	–0.189	0.010	–0.036	–0.055
Urban area	–0.044	–0.071*	–0.108*	0.004	–0.064	0.015	–0.061	0.009
European region	0.038	0.422*	0.072*	–0.139*	0.186*	–0.077*	–0.130*	–0.120*
High-risk scenario	0.000	0.018	0.056*	0.036	–0.056	–0.013	0.040	0.002
Risk for influenza	0.025*	0.004	0.012	0.009	–0.051	0.001	–0.006	0.014
Health	0.017	–0.009	–0.008	–0.013	–0.071*	–0.009	0.016	–0.011
Vaccinated	0.019	0.011	0.041	0.025	0.045	0.002	0.003	0.022
Employed	–0.051*	–0.059	–0.081*	–0.070*	0.053	–0.059	–0.033	–0.113*
Observation	1,341	1,263	1,355	1,307	349	1,293	1,310	1,316
Log likelihood	–710.699	–687.188	–843.241	–863.953	–226.204	–887.342	–744.488	–747.710

*Significant at 0.05.

In general, individual characteristics such as age, sex, self-reported influenza vaccination, and health status had little effect on reported precautionary measures (although younger persons were less likely to avoid places of entertainment and more likely to take leave from work). Even persons’ perceived risk for influenza had little effect except for avoiding public transportation; more respondents with higher risk perceptions reported being likely to avoid this setting (Table 4). The only individual-level variable that appeared to affect many of the precautionary actions was employment status. Fewer employed respondents reported being likely to avoid public transportation, entertainment venues, and work, and less likely to stay at home than those not employed full-time (e.g., homemakers, retirees, students). Although employed respondents were less likely (or perhaps less able) to adopt precautionary measures for themselves, they were more likely than persons who were not employed to report that they would withdraw their children from school (Table 4). The other notable individual-level covariate was education. In general, more respondents with higher educational levels reported being likely to avoid entertainment and shopping than did those with lower educational levels. Those with higher educational levels were generally less likely to report that they would take precautionary measures in other settings, but the effects were not statistically significant (Table 4).

The risk scenario given to the respondents (high vs. lower illness and death rates) did not significantly affect the results (Table 4). Region, however, did affect many of the reported precautionary actions. For example, Europeans were 19% more likely than Asians to report that they would keep their children from school, whereas Europeans were 13% less likely to report that they would avoid seeing physicians (Table 4). Respondents who lived in urban areas were less likely than their rural counterparts to report that they would avoid entertainment venues and restrict their shopping to the essentials, although the differences were not large (Table 4).

## Discussion

As a part of a large population-based survey of perceptions of pandemic influenza risk, we studied preventive behavior in 8 regions. Conducting comparable surveys in a number of different countries (3 of which had large SARS outbreaks in 2003) made it possible to make intercountry comparisons and assess underlying factors that may lead to precautionary actions. Our results suggest that large numbers of persons would try to take precautionary measures to reduce their risk of acquiring pandemic influenza. Approximately 75% of respondents said that they would avoid public transportation, and similar numbers would avoid places of entertainment and restrict their shopping to the essentials. These reported actions are in agreement with those reported in similar hypothetical studies and recorded behavior in the face of an epidemic. A recent survey of public health professionals in the United States (*16*) indicated that almost half would avoid work, a proportion similar to that reported by the general public in our survey. A survey of the Chinese community in the Netherlands, conducted just after the SARS epidemic, indicated that 84% had avoided travel to SARS-affected areas and 50% had avoided large gatherings of people (unpub. data), results that are comparable to those reported here. Furthermore, data on the use of public transportation and entertainment facilities in SARS-affected regions (*17*) suggest that demand for these services is affected by the public’s perceived risk of acquiring disease. The effect of such precautionary measures could be large in the case of pandemic influenza; the east and Southeast Asian economies lost an estimated $60 billion in the SARS outbreak because of reduced demand and business revenues (*18*).

Knowledge of what persons are likely to do can be used to estimate the health and economic effects of various pandemic influenza scenarios. We describe what proportion will take precautionary actions as well as the socioeconomic background of these persons, which would be useful for improving communication efforts by public health officials and clinicians in response to an outbreak.

One of the strengths of our study was its multicountry approach; with few exceptions, the patterns of potential precautionary actions were similar among respondents in each region. Public transportation was generally regarded as the most risky place and most likely to be avoided; home was regarded as the least risky setting. Individual-level characteristics such as age, sex, health, and educational status played little role in reported precautionary actions. Some regional differences were noted; Asian respondents reported that they were less likely to avoid restaurants and other entertainment establishments and more likely to avoid visiting physicians (the latter may have been related to their increased awareness of SARS [Table 3], which was often acquired in a healthcare setting). The identification of shops and hospitals as risky places had the largest variation between countries in Europe but the smallest variation between regions in Asia (Figure 1). The dominant pattern, however, was broadly similar across sociodemographic, health, and geographic strata.

The multivariate analysis provides some useful insights. Employment has emerged as an important determinant of prospective precautionary actions; employed persons were less likely to report that they would take preventive actions.

Our measure of risk perception (combining severity and vulnerability) was not associated with precautionary actions (except avoiding public transportation), and the measures of severity and vulnerability, separately, did not indicate any statistically significant influence. Thus, neither the risk perception score nor its individual components seemed to affect preventative actions, apart from the likelihood of avoiding public transportation. Liu et al. (*19*) found different components of risk perception to be significant in different geographic areas. In the Netherlands, higher risk perception was associated with more self-reported precautionary actions for SARS; however, when other explanatory variables like age, sex, education were included, no significant association between risk perception and precautionary actions was observed (*20*). If risk perceptions really do have little effect on precautionary behavior, public health messages aimed at changing persons’ perceptions of risk might be ineffective at changing their behavior. Clearly, this area requires further empirical study.

The main drawback of this type of survey is the difficulty in validating results. First, the participation rate varied 21%–81% among regions. Although a low response brings into question the representativeness of the samples, the similarity in findings between regions suggests that the low participation rate in some regions did not bias the findings. Second, because of the hypothetical nature of the questionnaire, concluding that persons actually would respond in the way that they have indicated here is not possible; however, the fact that a large section of the Dutch Chinese population did report taking precautionary actions to avoid SARS lends support to our findings (unpub. data).

Although the quantitative nature of the results may be difficult to validate, the qualitative findings are likely to be more robust. A new influenza pandemic would most likely result in persons’ limiting their use of public transportation, entertainment, and shopping for nonessentials. Also, although the public may perceive the risk from healthcare facilities to be relatively high, they would not necessarily avoid them.
